# The gut microflora assay in patients with colorectal cancer: in feces or tissue samples?

**Published:** 2019-02

**Authors:** Sama Rezasoltani, Hossein Dabiri, Hamid Asadzadeh-Aghdaei, Abbas Akhavan Sepahi, Mohammad Hossein Modarressi, Ehsan Nazemalhosseini-Mojarad

**Affiliations:** 1 Department of Biology, Science and Research Branch, Islamic Azad University, Tehran, Iran; 2 Department of Medical Microbiology, Faculty of Medicine, Shahid Beheshti University of Medical Sciences, Tehran, Iran; 3 Basic and Molecular Epidemiology of Gastrointestinal Disorders Research Center, Research Institute for Gastroenterology and Liver Diseases, Shahid Beheshti University of Medical Sciences, Tehran, Iran; 4 Gastroenterology and Liver Diseases Research Center, Research Institute for Gastroenterology and Liver Diseases, Shahid Beheshti University of Medical Sciences, Tehran, Iran

**Keywords:** Gut microbiota, Colorectal cancer, Fecal samples, Tissue specimens

## Abstract

Gut microbiota is the complex community of microorganisms that live in the digestive tracts of humans and other animals, including insects. The relationship between gut microbiota and human health is mutualistic and altered bacterial compositions in fecal and mucosal specimens of colon in patients with cancer compared to healthy subjects were observed. Thereby, studying the gut microbiota, their interactions with the host and their alterations in colorectal cancer (CRC) patients could be helpful to diagnose and treat the disease in earlier stages. In CRC research, the most common samples are feces and tumor tissues. Interestingly, scientists have quite different views regarding gut microbiota composition of feces and tissues. Some believe bacterial populations in feces and mucosa are completely distinct and differ in composition and diversity while some others declare similar variations. Actually, both types of specimens have some advantages and disadvantages in survey of gut microbiota. Fecal samples serve as a noninvasive approach for screening tests while mucosal associated samples are more powerful for identification of bacteria with adenoma and CRC initiation and growth. Here we have discussed the advantages and disadvantages of two type of specimens in CRC investigations and also discussed the similarities and differences of microbial composition between stool and tissue specimens.

## Colon cancer and gut microbiota

Colorectal cancer (CRC) is one of the most common causes of cancer-related mortality worldwide (1). Early diagnosis of this neoplasia is a critical step and may reduce patient mortality (2, 3). The international CRC subtyping consortium (CRCSC) re-analyzed 18 published datasets and identified four consensus molecular subtypes (CMS): CMS1 (MSI Immune, 14%), hypermutated, microsatellite unstable, strong immune activation; CMS2 (Canonical, 37%), epithelial, chromosomally unstable, marked WNT and MYC signaling activation; CMS3 (Metabolic, 13%), epithelial, evident metabolic dysregulation; and CMS4 (Mesenchymal, 23%), prominent transforming growth factor β activation, stromal invasion, and angiogenesis (4, 5). CRC is the third most common cancer worldwide after lung and prostate cancer in males and also is the second most common malignancy after breast cancer in females (6). During the past two decades, despite progress in chemotherapy and cancer control strategies, the survival rates of CRC patients have not changed, particularly in metastatic patients (7). Overall, prognosis, response to therapy and survival in CRC patients appear to demand stage of the tumor at the time of diagnosis and disease development (8). Most cases are often diagnosed when cancer is in advanced and uncontrollable stages. Hence, there is an urgent demand to identify and explore new biomarkers and reliable CRC diagnostic methods (9). Recently, the relationship between gut microbiota (the set of microorganisms that reside in the human gut) and CRC initiation and progression via the pro-carcinogenic activities of pathogens, especially metabolites and metabolite functions, have been debated (2, 3, 10). A role for gut microbiota in CRC growth was first proposed in germ-free mice almost 50 years ago, and the existence of disease-related bacteria (termed pathobionts) had increasing evidence from experimental data of microbial gavage, mono association or fecal transplantation (11).

Gut microbiota may be an important player in tumor initiation and progression, as cancer incidence in the large intestine is approximately 12-fold higher than the small intestine, which is attributed to greater bacterial density in the colon (10^12^ cells per ml) compared to (10^2^ cells per ml) the small intestine (12). Gut microbiota also affects other organs and systems such as cardiovascular system, lung, liver, bone and brain (13–15). Therefore, any imbalance in the healthy gut microflora or dysbiosismay result in several diseases like diabetes, obesity, metabolic syndrome, inflammatory bowel disease, irritable bowel syndrome, celiac disease and CRC (10, 16).

Gut microflora could derive miRNA and small non coding RNA (sncRNA) that signal between cells, tissues and also may be between bacterial species demonstrate that human being might be considerably influenced by the intestinal microbiota function which are regulated miRNA and sncRNA trafficking (17, 18). Also the interplay between gene methylation and gut microbiota in CRC has been identified. Gut bacteria could directly influence DNA replication, transcription, repair system, RNA splicing, and chromatin remodeling (19–21). Remarkably, gut microbiota up regulated some transcription factors involved in the regulation of the epithelial to mesenchymal transition, referred here as epithelial mesenchymal transition (EMT) in CMS classification. EMT is a cellular process that consists in the conversion of epithelial cell phenotype to a mesenchymal phenotype. Under physiological conditions, EMT is clearly critical for embryogenesis, organ development, wound repair and tissue remodeling (22). For example polysaccharide A (PSA) in *Bacteroides fragilis* inhibited CRC cell proliferation by controlling the cell cycle and impaired CRC cell migration and invasion by suppressing EMT (23).

By now, dietitians declare the importance of the probiotics in gut health that alter gut microbiota and lead to elaboration of gut flora metabolites which influence human health. So restoring the balance of intestinal flora by recommending probiotics for disease prevention and treatment might be beneficial. As with recent probiotics called next generation probiotics (NGP), one strategy involves associating the absence or presence of specific strains with a health phenotype and determining whether these chosen strains, when administered in sufficient quantities, can recapitulate the health phenotype (23, 25, 26).

Despite that there are still lots of difficulties and deficiencies related with utilizing gut microbiome in CRC therapy, gut microbiota-based CRC treatment is well tolerant, comparatively safe, and of a comfortable pattern. Combined application of gut microbiota and other therapeutics, particularly immunotherapy, display a powerful synergistic efficiency to restrain side effect and treat CRC. Clinical researches will help to get an appropriate understanding of molecular mechanism, which could further expand the application of gut microbiome in the early detection and prevention of CRC (27, 28). Taken together, fecal microbiota based approaches may provide additional methods for monitoring and optimizing anti-cancer treatments (28, 29).

## Fecal or mucosal biopsy/ resection samples in CRC?

The specimens used in CRC investigation are fecal or mucosal biopsy/ resection samples. Although some researchers believe that bacterial populations in feces and mucosa are completely distinct and differ in composition and diversity (30–32) because the composition of gut micrbiota adherent to the mucosal tissue and the fecal microbiota are depended on the oxygen gradient in the intestine and the nutrients provided by the host tissue (33), some others believe that similar variations in the frequency of CRC bacterial species can be detected between stool samples and patients biopsy (24, 34). Hence fecal samples findings may lead to conclusion on the metabolic and functional pattern of intestinal microbiota in the tumor microenvironment as well (24, 34, 35). While easily obtainable stool samples are important for developing tools for risk stratification and CRC screening compared to tissue samples (24, 36, 37) accordingly, by non-invasive fecal sampling and studying the changes in bacterial species associated to neoplasia, it might be possible to diagnose early-stages of neoplasia growth and detect the advanced adenomas such as CRC, however, widely screening of the pre-cancerous ulcers with high sensitivity in stool samples is still a big challenge (24). On the other hand it has proven that mucosal associated samples are important from a prevention stand point, as they allow for better identification of bacteria with adenoma and CRC initiation and growth (37). Castellarin et al. and Kostic et al. certainly explained fecal samples reflect the microbial composition in the tumor environment; however, profiling colonic tissue samples with shot gun metagenomic sequencing is still in effective due to excessive contamination with human DNA (38, 39). In agreement with Castellarin et al. and Kostic et al. a strong experiment has been done by Sobhani et al. resulting similar relative abundances of bacterial species between fecal and biopsy samples of CRC patients despite of different appearances in patient nationality, sample origin, experimental techniques and analysis methodology (24). In fact they sequenced 16S rRNA gene in 48 tumor-normal tissue pairs in terms to find if distinguish relevant differences between the microbial communities among tumor site and stool samples (24). Finally they observed fecal CRC marker species from the *Fusobacterium* genus showed a consistent enrichment at the tumor site, as was expected from Castellarin et al. and Kostic et al. (24, 38, 39). Sobhani et al. declared bacterial abundance differences in feces between CRC patients and tumor free controls were as well as between tumor and normal tissue. Also they demonstrated most metagenomic marker species with significantly decreased abundance in stool of CRC patients showed similar abundance changes in normal tissue compared to tumor, as it was the case for *Eubacterium* spp. and *Streptococcus salivarius* (24).

Moreover, reduction in the diversity of bacterial species in the intestinal microbial community is often related to an increase of colonization at the mucosal layer and the bacterial invasion to the epithelial layer in the active region of disease (3). This experience was exactly observed in both stool and tissues samples (40, 41). For instance increased abundance of *Fusobacterium, Porphyromonas, Coriobacteridae* and *Roseburia* (40, 42) and decreased abundance of *Firmicutes*, specifically *Clostridia* (involved in fermentation of dietary fiber (43) and *Enterobacteriaceaein* both stool and mucosal samples were achieved (32, 34).

Despite all mentioned above, each type of specimen has some advantages and disadvantages. Mucosal samples allow for better detection of bacteria and may be more specific in the stages of the disease so gut microbiota imbalance and interactions could be studied more directly. However there is distinct bacterial populations native to the proximal and distal sides of the colon (44). On the other, stool samples are easily obtainable and important for CRC screening (24). Detection of molecular biomarkers in fecal samples for the non-invasive early diagnosis of CRC may be more promising alternative than other biomarkers to be implemented in present clinical settings (45).

There are some variations in the CRC related bacteria found in different samples. Actually *Lactobacillales* enriched in CRC tissue, while *Fusobacterium, Porphyromonas, Peptostreptococcus, Gemella, Mogibacterium* and *Klebsiella* enriched in CRC mucosal adherent flora, also *Erysipelotrichaceae, Prevotellaceae* and *Coriobacteriaceae* enriched in the lumen of CRC patients (2, 46).

Overall besides clinical setting, *in vitro* studies are also recommended. However, type of the specimen, the complexity of gut microbiota actions, the relative abundance of each microbial species, the real spatial exposure of host cells to the microbial bodies or products and the human tissue complex interactions, make it hard to predict the CRC risk based on *in vitro* outcomes but it could be useful step for studying gut microbiota besides clinical studies. In addition, there are some technical limitations in distinction of the CRC marker bacteria, including limitations in conventional culture techniques, the expense of the sequencing technology, under powered cohort sizes, site and way of sample collection and processing, the method of DNA extraction technique, primers and reference sequence database quality (46). All of this requires bring us to focus more on clinical studies rather than *in vitro* settings.

## CONCLUSION

It was concluded although some researchers believe that bacterial populations in feces and mucosa are distinct and differ in composition and diversity, some others believe similar variations in the frequency of CRC bacterial species can be detected between stool and tissue samples. Each type of specimen has some advantages and disadvantages. Actually, survey of fecal samples as noninvasive approach could be useful for screening test of CRC. Also, the eukaryotic DNA contamination in fecal samples is less probable while mucosal associated samples are better and more powerful specimens for identification of bacteria with adenoma and CRC initiation and growth. Hence it might be more specific in all stages of the disease to identify gut microbiota imbalance and interactions directly. In this way it is more informative to consider the fecal and tissue samples in complementary. Finally besides clinical setting, *in vitro* studies of gut microbiota are also subscribed however some technical limitations including the complexity of gut microbiota actions, the relative abundance of each microbial species, the real spatial exposure of host cells to the microbial bodies or products and the human tissue complex interactions, make it hard to predict the CRC risk based on *in vitro* outcomes.
